# Ranging Behaviour of Commercial Free-Range Broiler Chickens 2: Individual Variation

**DOI:** 10.3390/ani7070055

**Published:** 2017-07-20

**Authors:** Peta S. Taylor, Paul H. Hemsworth, Peter J. Groves, Sabine G. Gebhardt-Henrich, Jean-Loup Rault

**Affiliations:** 1 Animal Welfare Science Centre, Faculty of Veterinary and Agricultural Sciences, University of Melbourne, Parkville, VIC 3010, Australia; phh@unimelb.edu.au (P.H.H.); raultj@unimelb.edu.au (J.-L.R.); 2 Poultry Research Foundation, School of Veterinary Science, Faculty of Science, The University of Sydney, Camden, NSW 2570, Australia; peter.groves@sydney.edu.au; 3 Research Centre for Proper Housing: Poultry and Rabbits (ZTHZ), Division of Animal Welfare, University of Bern, CH-3052 Zollikofen, Switzerland; sabine.gebhardt@vetsuisse.unibe.ch

**Keywords:** poultry, pasture, outdoor, range, meat chicken, welfare, Radio Frequency Identification (RFID)

## Abstract

**Simple Summary:**

Although the consumption of free-range chicken meat has increased, little is known about the ranging behaviour of meat chickens on commercial farms. Studies suggest range use is low and not all chickens access the range when given the opportunity. Whether ranging behaviour differs between individuals within a flock remains largely unknown and may have consequences for animal welfare and management. We monitored individual chicken ranging behaviour from four mixed sex flocks on a commercial farm across two seasons. Not all chickens accessed the range. We identified groups of chickens that differed in ranging behaviour (classified by frequency of range visits): chickens that accessed the range only once, low frequency ranging chickens and high frequency ranging chickens, the latter accounting for one-third to one half of all range visits. Sex was not predictive of whether a chicken would access the range or the number of range visits, but males spent more time on the range in winter. We found evidence that free-range chicken ranging varies between individuals within the same flock on a commercial farm. Whether such variation in ranging behaviour relates to variation in chicken welfare remains to be investigated.

**Abstract:**

Little is known about broiler chicken ranging behaviour. Previous studies have monitored ranging behaviour at flock level but whether individual ranging behaviour varies within a flock is unknown. Using Radio Frequency Identification technology, we tracked 1200 individual ROSS 308 broiler chickens across four mixed sex flocks in two seasons on one commercial farm. Ranging behaviour was tracked from first day of range access (21 days of age) until 35 days of age in winter flocks and 44 days of age in summer flocks. We identified groups of chickens that differed in frequency of range visits: chickens that never accessed the range (13 to 67% of tagged chickens), low ranging chickens (15 to 44% of tagged chickens) that accounted for <15% of all range visits and included chickens that used the range only once (6 to 12% of tagged chickens), and high ranging chickens (3 to 9% of tagged chickens) that accounted for 33 to 50% of all range visits. Males spent longer on the range than females in winter (*p* < 0.05). Identifying the causes of inter-individual variation in ranging behaviour may help optimise ranging opportunities in free-range systems and is important to elucidate the potential welfare implications of ranging.

## 1. Introduction

Broiler chicken ranging behaviour remains poorly understood, particularly on free-range commercial farms. A greater understanding of ranging behaviour can assist to ensure optimal opportunities to range through the provision of adequate environment and management practices, and possibly by selecting pertinent chicken characteristics. 

The majority of studies on broiler chicken ranging behaviour to date report variability in range use at flock level [1,2,3], which has been attributed to environmental conditions such as resources on the range (such as artificial and natural shelters, hay bales, perches and panels [4,5,6,7,8]) and weather variables (including outdoor temperature and Ultra Violet index [4,7,9]). Yet, very little is known about variation between individual broiler chickens within a flock. Genetics and rearing environments have been shown to alter ranging behaviour within a flock [6,10] but relationships with individual ranging behaviour is unknown. 

Heterogeneous ranging behaviour may result in variation in individual welfare and reduce uniformity in flocks. There are various beliefs that accessing an outdoor range will impact an animal’s welfare state; some consumers believe that accessing an outdoor range will have positive effects on broiler chicken welfare (e.g., increased expression of natural behaviours) and other groups (such as some farmers and veterinarians) are concerned with negative welfare consequences of range access, such as increased health risks due to increased exposure to parasites and extreme weather conditions [11,12]. However, there is very little scientific evidence of the impact of range access on broiler chicken welfare. Chicken welfare assessments are often reported as flock averages, however if variation in ranging behaviour exists then chickens within the same flock may have different welfare implications from ranging depending on the degree of variation. If welfare is compromised with increased range use, productivity (growth) may also be affected and result in reduced flock uniformity; an additional challenge for free-range flock management. 

In order to assess whether heterogeneous ranging behaviour exists in commercial broiler chicken flocks, we monitored individual broiler chicken ranging behaviour to determine the variation in ranging behaviour between individuals within commercial free-range flocks. 

## 2. Materials and Methods

All animals used in this study were approved by the University of Melbourne Animal Ethics Committee (approval number 1413428.3). A full description of the methodology is provided in part one of this paper series “Commercial free-range broiler chicken ranging behaviour 1: factors related to flock variability”; however, it is briefly outlined below.

### 2.1. Study Site and Animals

Four flocks (A–D) of ROSS 308 broiler chickens were studied across two seasonal replicates on one commercial farm during the Austral winter (flocks A and B) and summer (flocks C and D). All sheds had chicks from the same hatchery, same feed, same manager, and comparable management practices. Seasonal replicates occurred within the same sheds (Shed one: 40.5 m × 9.3 m, housing approximately 6000 chickens, flocks A and C; Shed two 50.5 m × 12.3 m, housing approximately 10,000 chickens, flocks B and D). Flocks had access to adjacent range areas (54.1 × 13.9 m and 77.9 × 16.4 m adjacent to the shed wall and 13.6 × 9.3 m and 27.5 × 12.3 m at the back of the shed, for shed one and two respectively) accessible through manually operated 1.3 × 0.4 m doors described hereafter as “pop-holes” and spaced 5.65 m apart, with six pop-holes for shed one and seven pop-holes for shed two. Feed and water were provided ad libitum inside the shed, but never in range areas. 

### 2.2. Tracking Individual Range Use

Individual range use was tracked by the Gantner Pigeon Radio Frequency Identification (RFID) System (2015 Gantner Pigeon Systems GmbH, Benzing, Schruns, Austria), with a bespoke program, Chicken Tracker. Chickens (*n* = 300/flock) were randomly selected and fitted with a silicone leg band that automatically loosened with leg growth (Shanghai Ever Trend Enterprise, Shanghai, China). Each leg band contained a unique ID microchip (Ø4.0/34.0 mm Hitag S 2048 bits, 125 kHz) that registered as the chickens walked over the antenna. Antennas were attached to both sides of each pop-hole (i.e., indoor and outdoor) to determine the direction of movements by each tagged chicken; allowing calculation of the frequency and duration of range visits for each individual.

Chickens were tracked from the first day that range access was permitted (21 days of age) until a few days before partial depopulation (30–33 days of age) in winter flocks due to logistical reasons. However, chickens in summer flocks were tracked until complete depopulation for slaughter (43–45 days of age). Sex and weight of individuals (flock A: *n* = 83, flock B: *n* = 97, flock C: *n* = 280, flock D: *n* = 290) were recorded at the end of the study when leg bands were removed. 

### 2.3. Statistical Analysis

RFID data were cleaned with SAS^TM^ (v. 9.3, SAS Institute Inc., Cary, NC, USA) using a modified macro [13]. All range visits <10 s were treated as false positives and removed from analysis. 

Descriptive data are presented for each flock.

Statistical analysis was performed with SPSS statistical software (v. 22, IBM Corp, Armonk, NY, USA). 

Latency to access the range data did not meet the criteria of normality; therefore, Spearman’s rho correlation coefficients were used to examine relationships between latency to first access the range and total frequency and duration of range visits and duration per range visit, relationships between total time spent on the range, total number of range visits and average time spent on the range per range visit. Chi square analysis was used to determine if there was a difference in the number of females and males that accessed the range. Flock could not be included as a random variable in non-parametric Spearman correlations or chi square analysis, but each correlation and chi square analysis was initially performed on each flock and there were no differences in direction or significance values between flocks within season. Hence, flocks were pooled and are presented within season. Ranging data were log transformed and subsequently met the criteria of normality and homogeneity of variance; hence, General Linear Mixed models were used to determine the effect of sex on the total frequency and duration of range visits, average time spent on the range per visit and the number of days an individual accessed the range, with flock and individual nested within flock as random factors, in addition to running the model both with and without final body weight as covariate. General Linear Mixed models were used to compare the average time spent on the range per range visit between chickens that accessed the range only once and chickens that accessed the range more than once, with flock and individual nested within flock included as random factors and weight as a covariate. Results are presented as raw means ± SE unless otherwise noted.

## 3. Results

### 3.1. Range Availability

Winter flocks had fewer opportunities (days and hours per day) to access the range than summer flocks; data were presented in part one of this paper series “Commercial free-range broiler chicken ranging behaviour 1: factors related to flock variability”. Briefly, in winter, management provided the flocks with access to the range for a mean of 5.6 ± 0.4 h a day, for 70% of the days prior to partial depopulation. In summer, management provided the flocks with access to the range for a mean of 10.4 ± 0.6 h a day, for 75% and 76.2% of the days prior to complete depopulation, in flocks C and D respectively. 

### 3.2. Inter-Individual Variation in Ranging Behaviour

**Figure 1:**
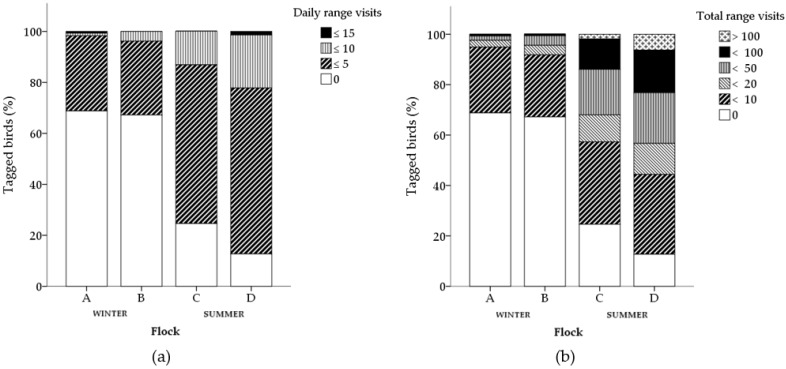
Frequency of range visits for individual chickens within each flock (winter flocks: A and B; summer flocks: C and D). Patterns within stacked bars represent the number of chickens (% successfully tracked) in each ranging frequency category, daily mean (**a**) and total number of visits throughout the study (**b**) for each flock.

**Figure 2:**
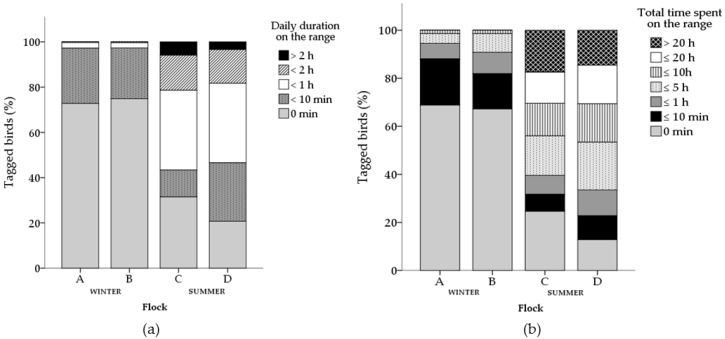
Duration of range visits for individual chickens within each flock (winter flocks: A and B; summer flocks: C and D). Patterns within stacked bars represent the number of chickens (% successfully tracked) in each ranging duration category, daily mean (**a**) and total time spent on the range throughout the study (**b**) for each flock.

There was individual variation in ranging frequency and duration within all flocks (Figure 1 and Figure 2). The mean number of daily visits made by an individual varied between 0–11.8 and 0–12.7 visits in winter and summer flocks, respectively. The mean time an individual spent on the range daily varied between 0–76.6% and 0–65.7% of the available ranging time, equivalent to 0–4.3 h and 0–6.8 h, in winter flocks and summer flocks respectively. 

The total number of range visits made by an individual varied between 0–71 and 0–167 visits over the course of the study in winter flocks and summer flocks respectively. The total duration an individual spent on the range over the course of the study varied between 0–23.0% and 0–40.2% of available overall ranging time, equivalent to 0 to 8.7 h and 0 to 40.7 h, in winter flocks and summer flocks respectively.

### 3.3. Latency to Access the Range

The number of days before an individual first accessed the range after range access was first provided (hereafter referred to as “latency to access the range”) was negatively correlated with an individual’s total number of range visits (winter: *r*_(188)_ = −0.41, *p* < 0.001; summer: *r*_(460)_ = −0.44, *p* < 0.001) and total duration of range visits (winter: *r*_(188)_ = −0.34, *p* < 0.001; summer: *r*_(460__)_ = −0.35, *p* < 0.001), but not the mean duration per visit. Latency to access the range was also negatively correlated with the number of days an individual accessed the range in summer flocks (*r*_(460)_ = −0.33, *p* < 0.01), but not in winter flocks. 

When individual ranging data were corrected for number of available ranging days remaining after the range was first accessed, to assess ranging patterns after first range access, latency to access the range was still negatively correlated with range use in both seasons (frequency: winter—*r*_(143)_ = −0.24, *p* < 0.01; summer—*r*_(450)_ = −0.34, *p* < 0.00; duration: winter—*r*_(143)_ = −0.20, *p* < 0.05; summer—*r*_(450)_ = −0.31, *p* < 0.001).

### 3.4. High Frequency Ranging Chickens

**Figure 3:**
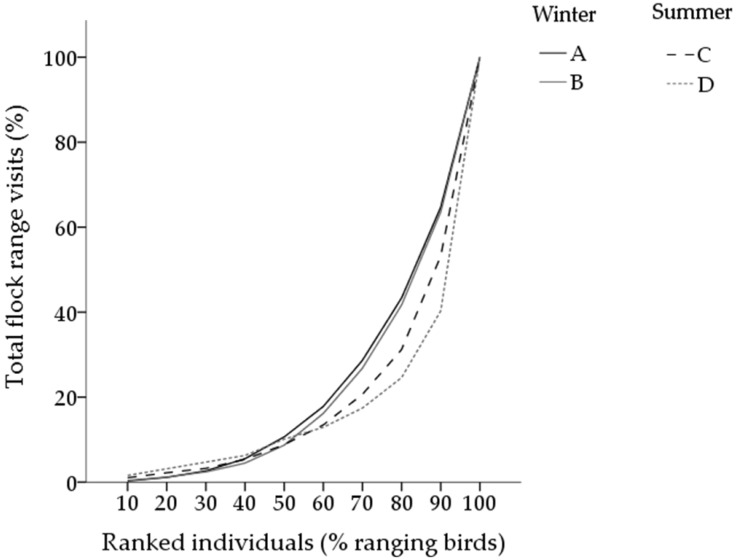
The proportion of range visits (% total flock range visits) that was attributed to ranked individuals. Ranging chickens were ranked on the total number of range visits and are displayed from lowest to highest percentage of ranging chickens in each flock; solid lines represent winter flocks (flocks A and B), dotted lines represent summer flocks (flocks C and D). Chickens that did not access the range are not included.

A total of 1434 range visits were recorded in winter flocks (flock A: 573 visits; flock B: 861 visits) and 14,008 range visits in summer flocks (flock C: 5644 visits; flock D: 8364 visits). The top 10% of ranging chickens, based on frequency of range visits, accounted for approximately half of the range visits in winter flocks (flock A: 9 chickens accounted for 57% total range visits; flock B: 10 chickens accounted for 47% total range visits) and one-third of range visits in summer flocks (flock C: 21 chickens accounted for 34% of range visits; flock D: 25 chickens accounted for 33% total range visits). The top 50% of ranging chickens accounted for 89–91% of all range visits, irrespective of season (Figure 3). Thus, the bottom 50% of ranked ranging chickens accounted for <15% of the total range visits (winter: flock A—13.3% of total visits, flock B—13.5% of total visits; summer: flock C—4.8% of total visits, flock D—5.9% of total visits).

The top 10% of ranging chickens, based on the total time spent on the range, accounted for more than half of the total flock time spent on the range in winter flocks (flock A: 70%, flock B: 54%) and more than one-third of the total flock time spent on the range in summer flocks (flock C: 38%, flock D: 37%). The average time spent on the range per visit did not differ between birds ranked in the top 10%, top 11–49% or bottom 50% in either season (winter: F_(2,184)_ = 0.4, *p* > 0.05; summer: F_(2,456)_ = 0.3, *p* > 0.05).

### 3.5. Chickens that Accessed the Range Once

There was a relatively consistent proportion of chickens, across all flocks, that accessed the range only once throughout the study (flock A: 12%, flock B: 10%, flock C: 6%, flock D: 8%). The total number of one-time ranging chickens on a particular day was positively correlated with the number of chickens on the range daily in summer flocks (*r_(_*_30)_ = 0.49, *p* < 0.05), but not in winter flocks. Conversely, the total number of chickens that ranged only once was positively correlated with age in winter flocks (*r*_(11)_ = 0.63, *p* < 0.05), but not in summer flocks.

Chickens that accessed the range only once spent longer on the range during that visit than the average time per visit by chickens that accessed the range more than once in summer flocks (one-time ranging chickens: 31.3 ± 12.4 min/visit, more than once ranging chickens: 22.4 ± 4.3 min/visit; F_(1,457)_ = 11.5, *p* < 0.01), but there was no difference in winter flocks (one-time ranging chickens: 9.9 ± 4.7 min/visit, more than one-time ranging chickens: 6.6 ± 0.9 min/visit; F_(1,65)_ = 3.46, *p* > 0.05).

### 3.6. Individual Ranging Variation and Relationships with Sex

The proportion of females and males that accessed the range did not differ (winter flocks: female ranging chickens—52.9%; male ranging chickens—47.1%; χ^2^_(1,180)_ = 1.43, *p* > 0.05; summer flocks: female ranging chickens—57.3%; male ranging chickens—42.7%; χ^2^_(1, 437)_ = 0.05, *p* > 0.05). The number of days that males and females accessed the range did not differ (winter flocks: females—3.7 ± 0.4 days; males—3.3 ± 0.3 days; χ^2^_(1, 65)_ = 0.46, *p* > 0.05; summer flocks: females—7.6 ± 0.3 days, males—7.0 ± 0.3 days; χ^2^_(1, 437)_ = 2.09, *p* > 0.05). 

The overall frequency of range visits did not differ between males and females in both seasons (winter flocks: female—18.8 ± 3.2 visits, male—12.8 ± 2.6 visits; summer flocks: female—35.3 ± 2.3 visits; male—24.3 ± 2.0 visits; all *p* > 0.05). Noteworthy, when weight was not included in the analysis females (lighter in weight than males) accessed the range more frequently than males in summer flocks (F_(1, 442)_ = 8.18, *p* < 0.01) but not winter (F_(1, 66)_ = 1.26, *p* > 0.05).

Males spent longer on the range overall than females in winter flocks (females: 2.0 ± 0.4 h, males: 2.3 ± 0.4 h; F_(1, 66)_ = 3.92, *p* = 0.052) but not summer (females: 11.8 ± 0.9 h, males: 9.5 ± 0.8 h; F_(1, 442)_ = 1.14, *p* = 0.29). Males spent longer on the range per range visit than females in winter (females: 12.7 ± 7.1 min, males: 17.3 ± 5.7 min; F_(1, 65)_ = 5.8, *p* < 0.05) but not summer (females:20.3 ± 1.2 min, males: 27.1 ± 2.5 min; F_(1, 442)_ = 0.47, *p* > 0.05). 

## 4. Discussion

Our results showed that ranging behaviour varied greatly between individuals within flocks from the same hatchery, genetic lines, feed composition and availability, management regime, stock people and environmental and range conditions. In all flocks, not all chickens accessed the range and the number of visits and time spent on the range varied greatly between individuals. Although the data clearly identifies a continuum of ranging variation, we have categorized chickens in this paper for simplicity and acknowledge that such categories are arbitrary. We observed chickens that accessed the range only once, high frequency ranging chickens that accounted for one-third to half of all of the range visits (depending on season) and low frequency ranging chickens ranked in the bottom 50% of all tagged chickens that ranged but accounted for less than 15% of all range visits throughout the study. 

The variation in ranging behaviour may reflect differences in the motivation to access the range. The high frequency ranging chickens accessed the range more frequently but also for a longer period of time overall and sooner after range access was first provided. High frequency ranging chickens have also been reported in commercial laying hens [13,14] and may be of particular interest to industry and consumers. Consumers that support free-range products often feel betrayed with reports of low range use in commercial flocks, leading to controversy and revised labelling regulations of free-range egg products in Australia for instance [15]. Determining the characteristics that result in high frequency range use may permit early life environmental interventions or breeding programs to encourage range use. A thorough understanding of such interventions is critical and the appropriate application will depend on the characteristics involved and the outcomes on the welfare of the chicken. 

Variation in ranging behaviour between individuals may also reflect individual experiences on the range. The most interesting group in this regard is the chickens that accessed the range only once throughout the study (6–12% of tracked chickens). Although the sample size of this group was low within each flock, it was relatively similar between flocks. One-time ranging chickens were not necessarily “accidental” range users, because the duration of range visits was greater for chickens that accessed the range once compared to those that accessed the range more than once in summer flocks. Perhaps the first range visit was a frightening experience for these chickens, which may have discouraged the chicken from going out again. Indeed, there are reports of links between exposure to a range environment and fearfulness in broiler chickens [16] and the number of days an individual visits the range and fearfulness in laying hens [17,18,19]. However, the direct relationship between fearfulness and individual broiler chicken ranging behaviour is unknown. It would be interesting to investigate the ranging experience of these particular chickens that accessed the range only once, such as the individual’s location, behaviour and environmental stimuli on the range during this single visit. 

Our results demonstrated that it is important to disentangle the effects of sex and weight on ranging behaviour of broiler chickens. Females and males did not differ in their ranging frequency when weight was included in the analysis, as lighter chickens accessed the range more frequently. This suggests that weight should always be included when comparing sex effects on ranging behaviour in broiler chickens, given the marked sexual-dimorphic growth of broiler chickens. Our findings differ from Chapuis, Baudron, Germain, Pouget, Blanc, Juin and Guemene [2] who monitored a slower growing strain of broiler chicken and conversely found that males made up a higher percentage of the top ranging chickens (60%) and females made up the majority of the lowest ranging chickens (70%). Although Chapuis, Baudron, Germain, Pouget, Blanc, Juin and Guemene [2] did not control for weight, sexually dimorphic contrasts are greater in slow growing broiler than fast growing strains [20] and it is likely that the difference in findings between the two studies would be exacerbated if growth was controlled for in their study. We hypothesize that our and Chapuis, Baudron, Germain, Pouget, Blanc, Juin and Guemene [2] findings may reflect temperament differences between sexes of chickens. Independent of weight, we found that males spent more time on the range overall and per visit than females, in winter flocks. Hence, sex characteristics other than weight may be associated with ranging behaviour such as those reported in strains of laying chickens, including fearfulness, exploratory behaviour or social behaviour [21,22].

We found a high level of variation in range use between seasons and within flocks. These findings highlight the importance of monitoring individual chickens when investigating relationships between range access and welfare. For example, if we were to measure a welfare indicator on our winter flocks, the likelihood of obtaining a measure from an animal that accessed the range at least once would have been only 33%, and a low 10% chance that the chicken would have accessed the range frequently. Clearly, there is a need to determine individual ranging patterns to understand the welfare implications of range use. In addition, the welfare implications of range restriction (during periods of extreme weather conditions or prior to depopulation, a typical commercial practice) on the behaviour and welfare of chickens that differ in their ranging behaviour and motivation remains to be elucidated.

This study provides details of individual variation in the ranging behaviour of broiler chickens on a commercial free-range farm. However, this study was only conducted on one broiler strain and one farm, and the external validity of the findings to other broiler strains, geographical areas, and farms with different flock sizes and range design is unknown. Further investigation is needed to determine the causal factors for this variation, since variation was observed between individuals in the same flock, with the same breeding and hatching history, same shed and range design and similar management practices. This knowledge could lead to science-based improvements in ranging opportunities of commercial free-range broiler chickens. 

## 5. Conclusions

Ranging behaviour varied between individuals within the same commercial flocks, revealing chickens that never accessed the range, chickens that accessed the range only once, low frequency ranging chickens, and high frequency ranging chickens, with the latter accounting for a third to a half of all range visits. Males spent more time on the range than females in winter flocks, but frequency of range visits was related to weight rather than sex in summer flocks.

These findings suggest that individual characteristics and/or early life experience partly determine ranging behaviour in commercial conditions, which subsequently results in heterogeneous flock ranging behaviour. The causes for this inter-individual variation in ranging behaviour within flocks should be investigated to ensure that chickens in free-range systems are best suited to such housing conditions and thus facilitate optimal ranging behaviour on commercial farms.
